# Global burden and prediction study of cutaneous squamous cell carcinoma from 1990 to 2030: A systematic analysis and comparison with China

**DOI:** 10.7189/jogh.14.04093

**Published:** 2024-05-03

**Authors:** Shudai Huang, Jiayi Jiang, Hoi-shiwn Wong, Ping Zhu, Xiang Ji, Daguang Wang

**Affiliations:** Department of Dermatology, The First Affiliated Hospital with Nanjing Medical University, Nanjing, Jiangsu, China

## Abstract

**Background:**

China has the highest number of new cancer cases and deaths globally. Due to particularly low scores in health care quality for cutaneous squamous cell carcinoma (cSCC), the country’s cSCC burden requires greater awareness. Consequently, we aimed to evaluate and predict the trend of the cSCC burden globally and in China from 1990 to 2030.

**Methods:**

We retrieved data from the Global Burden of Disease 2019 study, which provided estimates of the incidence, mortality, prevalence, and disability-adjusted life years (DALYs) of cSCC from 1990 to 2019. We set up joint-point analyses and Bayesian age-period-cohort (BAPC) models to predict the disease burden of cSCC up to 2030.

**Results:**

In 2019, China reported age-standardised rates of cSCC prevalence, incidence, mortality, and DALYs of 2.54, 2.12, 0.88, and 16.76 per 100 000 population, respectively. The country’s prevalence and incidence rates from 1990 to 2019 were lower than the global levels, but its mortality and DALY rates were higher. The age-standardised rates were higher for males, and the disease burden increased with each age group globally and in China. Moreover, the average annual percentage change showed all indicators were growing faster than the global levels. According to the BAPC model, there will be an upward trend in the prevalence and incidence globally and in China between 2020 and 2030, with a decrease in mortality and DALYs.

**Conclusions:**

We observed an upward trend in the cSCC burden over the past 30 years in China. Prevalence and incidence are expected to continue at a higher rate than the global average in the next decade, while mortality and DALYs are predicted to decrease. As the Chinese population ages, efforts toward managing and preventing cSCC should be targeted towards the elderly population.

Cutaneous squamous cell carcinoma (cSCC) is the second most prevalent skin tumour, with a growing incidence attributed to multiple factors, including exposure to ultraviolet (UV) light, genetic predisposition, and environmental elements [1]. It is known to be invasive and prone to metastasis. In 2019, the global age-standardised prevalence of cSCC was 39.29 per 100 000 population, the highest among the three common skin cancers, including malignant skin melanoma (25.97 per 100 000 population) and basal cell carcinoma (5.15 per 100 000 population) [2].

Moreover, research has found that the recurrence of cSCC following surgery ranges from 3.5% to 28.0% [3], creating medical challenges and economic burdens. In fact, skin cancer has been identified as the fifth most expensive cancer to treat [4], with costs associated with melanoma and non-melanoma skin cancers in the USA ranging from USD 39.2 million to USD 28.9 million for morbidity and from USD 3.3 billion to USD 1.0 billion for mortality, based on a systematic review of articles published between 1990 and 2009 [5]. Despite this, our understanding of cSCC remains lacking, as studies on its epidemiology had mostly analysed it in combination with other skin tumours. Factors including the ageing population, climatic changes, and economic fluctuations may influence the burden [42,44,45].

While reports have analysed the global burden of cSCC, none have explored it in China specifically. China is a developing country with a population of 1.4 billion. According to the GLOBOCAN 2020 study, it has the highest number of new cancer cases and deaths globally [6]. Moreover, based on data from the Global Burden of Disease (GBD) study, China scored low on the Healthcare Access and Quality (HAQ) index for cSCC compared to other countries [7]. Consequently, understanding the cSCC burden in China would allow for an improved distribution of resources and the development of evidence-based health care policies. Moreover, findings on trends of cSCC in China may be relevant for other areas with low incidence, limited resources, or a lack of reliable disease burden data. Seeing the potential relevance of such research, we analysed the GBD 2019 data to determine and predict the trends of the cSCC burden in China from 1990 to 2030, and to compare them to the global level.

## METHODS

### Data sources

We retrieved data from the GBD 2019 database, which provided estimates of the incidence, mortality, prevalence, and disability-adjusted life years (DALYs) associated with 369 illnesses and injuries from 1990 to 2019 [8–10]. This included annual age-specific data on cSCC prevalence, incidence, deaths and DALYs as absolute numbers, crude rates, and age-standardised rates (ASRs) with 95% confidence intervals (CIs) for the 1990–2019 period for both China and the world; age-specific population data for China and the world from 1990 to 2019; and projected population data from 2020 to 2030.

### Descriptive analysis

We described the temporal trends of the burden of cSCC both globally and in China from 1990 to 2019 and stratified the analyses by age groups, gender, and the socio-demographic index (SDI), a composite indicator of per capita income, years of education, and the fertility rate of the population under 25 years of age, with larger values representing higher levels of development. We estimated ASRs using the GBD world standard population as a reference [11].

### Joinpoint regression analysis

We used joinpoint regression models to evaluate the temporal trends of the burden of cSCC from 1990 to 2019 by calculating average annual percentage change (AAPC). These models break the data into segments and fit linear regression within each segment, effectively capturing trend changes. The core principle involves finding the optimal segmentation to match observed trends best, making it useful for analysing pattern shifts. Here we used Joinpoint, version 4.9.1. (National Cancer Institute, Rockville, MD, USA) to fit the simplest joinpoint model allowed by the data (i.e. the model with the fewest number of connection points) and used the most optimal one to calculate the AAPC [12]. The regression line equation can be expressed as *y* = α + *βx* + ε, where *y* = *ln*(*ASR*) and *x* = calendar year. Meanwhile, the AAPC is calculated as *100* × (*exp*(β) − *1*) [13], with values >0 indicating an increasing trend and those <0 indicating a decreasing trend. We considered a *P*-value <0.05 as statistically significant.

### Construction of Bayesian age-period-cohort model

The Bayesian age-period-cohort (BAPC) model is a statistical tool that helps researchers understand trends such as disease rate changes. It focusses on three aspects: The age (i.e. an individual’s age), period (i.e. external factors affecting everyone), and cohort (i.e. people born in the same timeframe). While traditional models need help with these intertwined factors, the BAPC model untangles their effects using Bayesian methods. Combining prior beliefs with observed data clarifies how age, period, and cohort impact outcomes while expressing the uncertainty in these estimates. Mathematically, the model can be represented as:

*y*(*a*,*p*,*c*) = α(*a*) + β(*p*) + γ(*c*) + *ɛ*(*a*,*p*,*c*)

where *y*(*a*,*p*,*c*)  is the observed outcome at a specific combination of age (*a*), time period (*p*), and birth cohort (*c*); α(*a*), β(*p*), and γ(*c*) are the age, period, and cohort effects, respectively; and *ɛ*(*a*,*p*,*c*) is the residual error term [14,15].

To estimate the effects, the BAPC model applies Bayesian inference, incorporating prior knowledge and updating it with the observed data to obtain posterior distributions, which are commonly explored through Markov Chain Monte Carlo sampling techniques [16,17]. Ultimately, the optimal model is selected by the deviance information criterion (DIC).

Based on age-specific population data from 1990 to 2019, projected population data between 2020 and 2030, and the GBD world population age standard, we used the BAPC model to predict the burden of cSCC from 2020 to 2030. We assumed our predictions of cSCC burden based on past trends, not considering changes in risk factors and interventions. We built the BAPC models in the ‘INLA’ and ‘BAPC’ packages of R, version 4.3.1 (R Core Team, Vienna, Austria).

### Sensitivity analyses

To validate the reliability of the BAPC model, we used cSCC burden of disease data from 1990–2010 for modelling to predict the burden of cSCC globally and in China from 2011 to 2019. Ultimately, we calculated the coefficient of determination (R^2^) by combining the observed values from 2011 to 2019.

## RESULTS

### Temporal trends in disease burden of cSCC in different regions from 1990 to 2019

**Figure 1:**
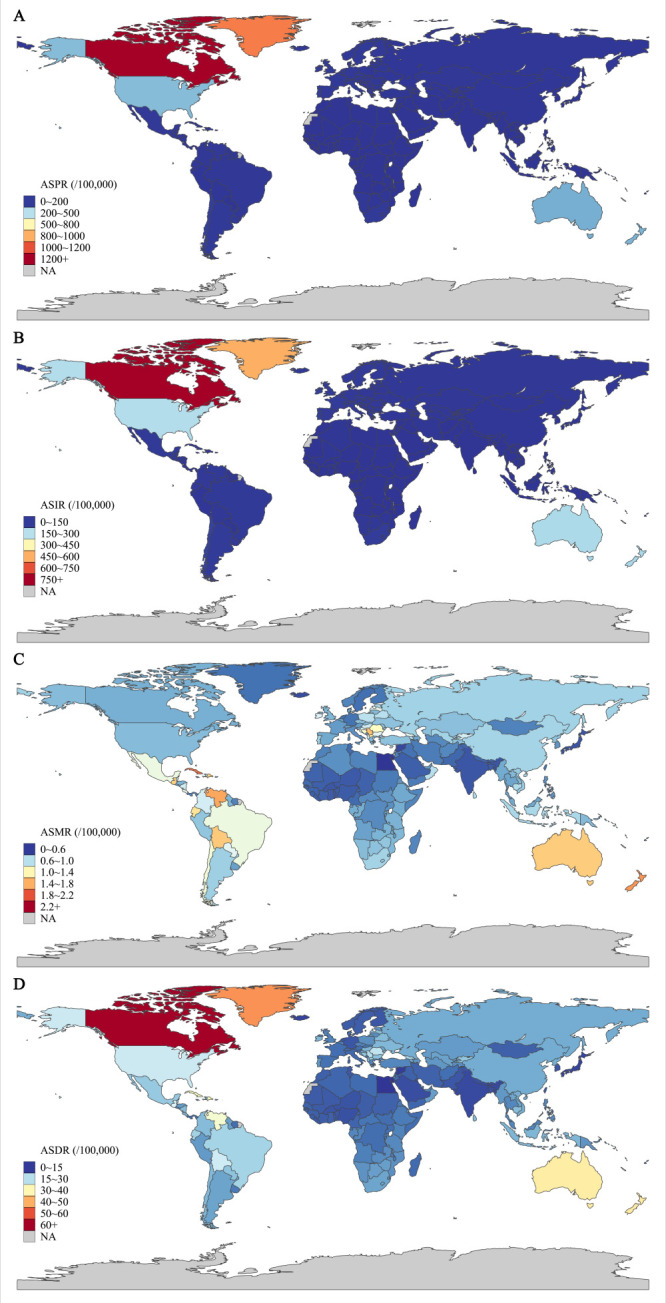
The distribution of ASIR, ASPR, ASMR and ASDR for cSCC in 204 countries and territories in 2019. **Panel A.** Regional distribution of the ASPR. **Panel B.** Regional distribution of the ASIR. **Panel C.** Regional distribution of the ASMR. **Panel D.** Regional distribution of the ASDR. ASDR – age-standardised disability-adjusted life years rate, ASMR – age-standardised mortality rate, ASIR – age-standardised incidence rate, ASPR – age-standardised prevalence rate.

**Figure 2:**
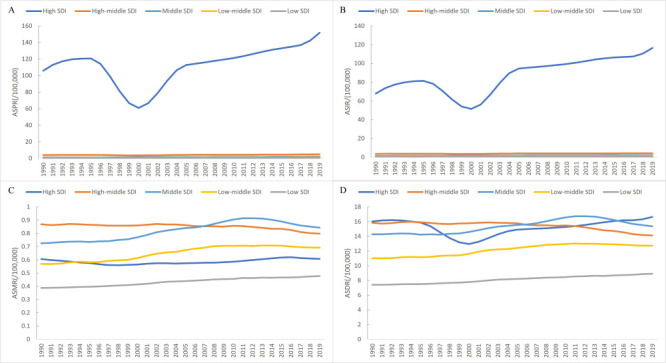
The distribution of ASIR, ASPR, ASMR and ASDR for cSCC by quintile of SDI. **Panel A.** ASPR by quintile of SDI. **Panel B.** ASPR by quintile of SDI. **Panel C.** ASIR by quintile of SDI. **Panel D.** ASIR by quintile of SDI. ASDR – age-standardised disability-adjusted life years rate, ASMR – age-standardised mortality rate, ASIR – age-standardised incidence rate, ASPR – age-standardised prevalence rate, SDI – sociodemographic index.

In 2019, high-income North America and Australasia had the highest age-standardised prevalence rate (ASPR), age-standardised incidence rate (ASIR), age-standardised mortality rate (ASMR), and age-standardised DALYs rate (ASDR) of the cSCC burden. In contrast, the disease burden from cSCC in most regions of Asia and Africa was relatively low (**Figure 1**; Table S1 in the **Online Supplementary Document**). Meanwhile, the ASIR and ASPR were substantially higher in areas with a high SDI; however, the regional differences in ASMR and ASDR were insignificant (**Figure 2**). Globally, the ASPR, ASIR, ASMR, and ASDR of cSCC were 39.29, 30.30, 0.73, and 14.64 per 100 000 population in 2019, respectively. The joinpoint regression analysis showed that the ASIR and ASMR of cSCC were rising, with AAPCs of 0.92 (95% CI = 0.42, 1.42) and 0.20 (95% CI = 0.11, 0.28), respectively. In comparison, the ASPR and ASDR of cSCC at the global level were relatively stable (**Table 1**).

**Table 1:** Change of age-standardised rates in prevalence, incidence, mortality, and DALYs for cSCC per 100 000 population between 1990 and 2019 in the world and in China

	Global				China			
	**ASRs in 1990, (95% UI)**	**ASRs in 2019, (95% UI)**	**AAPC (1990–2019), % (95% CI)**	***P*-value**	**ASRs in 1990, (95% UI)**	**ASRs in 2019, (95% UI)**	**AAPC (1990–2019), % (95% CI)**	***P*-value**
Prevalence	34.44 (28.09, 42.00)	39.29 (33.96, 45.77)	0.26 (−0.40, 0.93)	0.43	0.24 (0.20, 0.29)	2.54 (2.14, 3.00)	8.51 (8.14, 8.89)*	<0.05
Incidence	22.27 (18.04, 27.43)	30.30 (26.89, 34.08)	0.92 (0.42, 1.42)*	<0.05	0.80 (0.69, 0.92)	2.12 (1.79, 2.48)	3.40 (3.21, 3.58)*	<0.05
Mortality	0.69 (0.63, 0.73)	0.73 (0.65, 0.78)	0.20 (0.11, 0.28)*	<0.05	0.70 (0.61, 0.78)	0.88 (0.74, 1.01)	0.75 (0.58, 0.91)*	<0.05
DALYs	14.42 (13.30, 15.40)	14.64 (13.43, 15.64)	0.03 (−0.04, 0.11)	0.36	14.60 (12.82, 16.48)	16.76 (14.17, 19.37)	0.43 (0.25, 0.61)*	<0.05

AAPC – average annual percentage change, ASR – age-standardised rate, CI – confidence interval, cSCC – cutaneous squamous cell carcinoma, DALYs – disability-adjusted life years, UI – uncertainty interval

*P < 0.05.

In China, the ASPR, ASIR, ASMR, and ASDR of cSCC in 2019 were 2.54, 2.12, 0.88 and 16.76 per 100 000 population, respectively. Moreover, the ASPR and ASIR were substantially lower than the global levels in 1990 and 2019, while the ASRs of the cSCC burden indicators kept increasing, with AAPCs were 8.51 (95% CI = 8.14, 8.89) for ASPR, 3.40 (95% CI = 3.21, 3.58) for ASIR, 0.75 (95% CI = 0.58, 0.91) for ASMR, and 0.43 (95% CI = 0.25, 0.61) for ASDR, which were all growing faster than the global levels (**Table 1**).

### Temporal trends in disease burden of cSCC in different gender from 1990 to 2019

**Figure 3:**
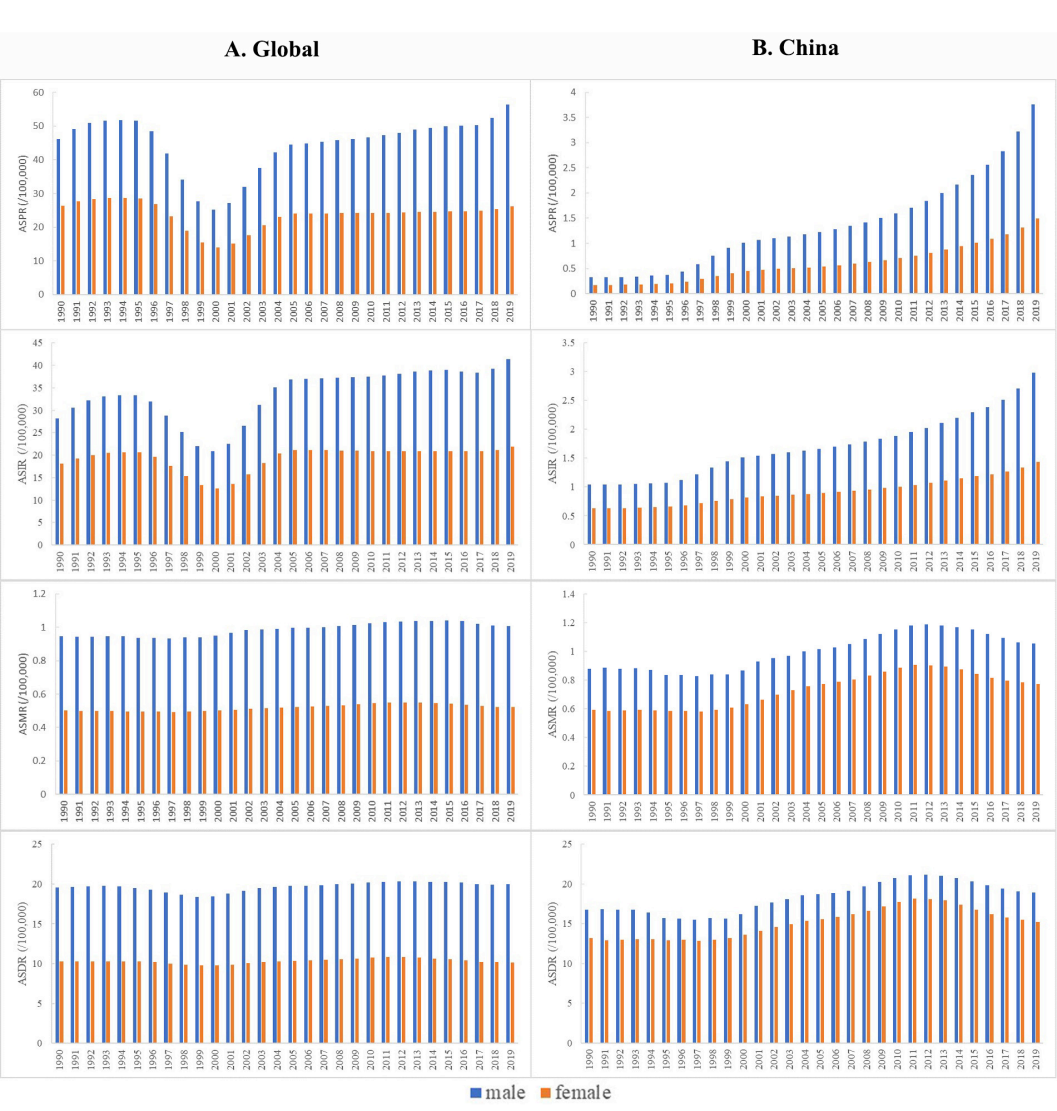
The gender distribution of ASPR, ASIR, ASMR and ASDR for cSCC from 1990 to 2019 at both global and China levels. **Panel A.** ASPR, ASIR, ASMR and ASDR for cSCC worldwide. **Panel B.** ASPR, ASIR, ASMR and ASDR for cSCC in China. ASDR – age-standardised disability-adjusted life years rate, ASMR – age-standardised mortality rate, ASIR – age-standardised incidence rate, ASPR – age-standardised prevalence rate.

Globally, the ASRs of cSCC burden indicators for men from 1990 to 2019 were higher and increased at more than double the rate than those for women. Moreover, the ASPR and ASIR of cSCC for men peaked in 2019 at 56.42 and 41.39 per 100 000 population, respectively, while their ASMR and ASDR peaked in 2015 and 2013 at 1.04 and 20.32 per 100 000 population, respectively. Additionally, the ASPR and ASIR of cSCC for men and women progressively declined to the lowest level in 2000 and kept increasing until 2019 (**Figure 3**).

In China, the burden of cSCC was also higher for men than women. The ASPR and ASIR of cSCC for men peaked in 2019 at 3.76 and 2.98 per 100 000 population, respectively, while the ASMR and ASDR of cSCC for men peaked in 2012 at 1.19 and 21.18 per 100 000 population, respectively (**Figure 3**).

### Temporal trends in disease burden of cSCC in different age group from 1990 to 2019

**Figure 4:**
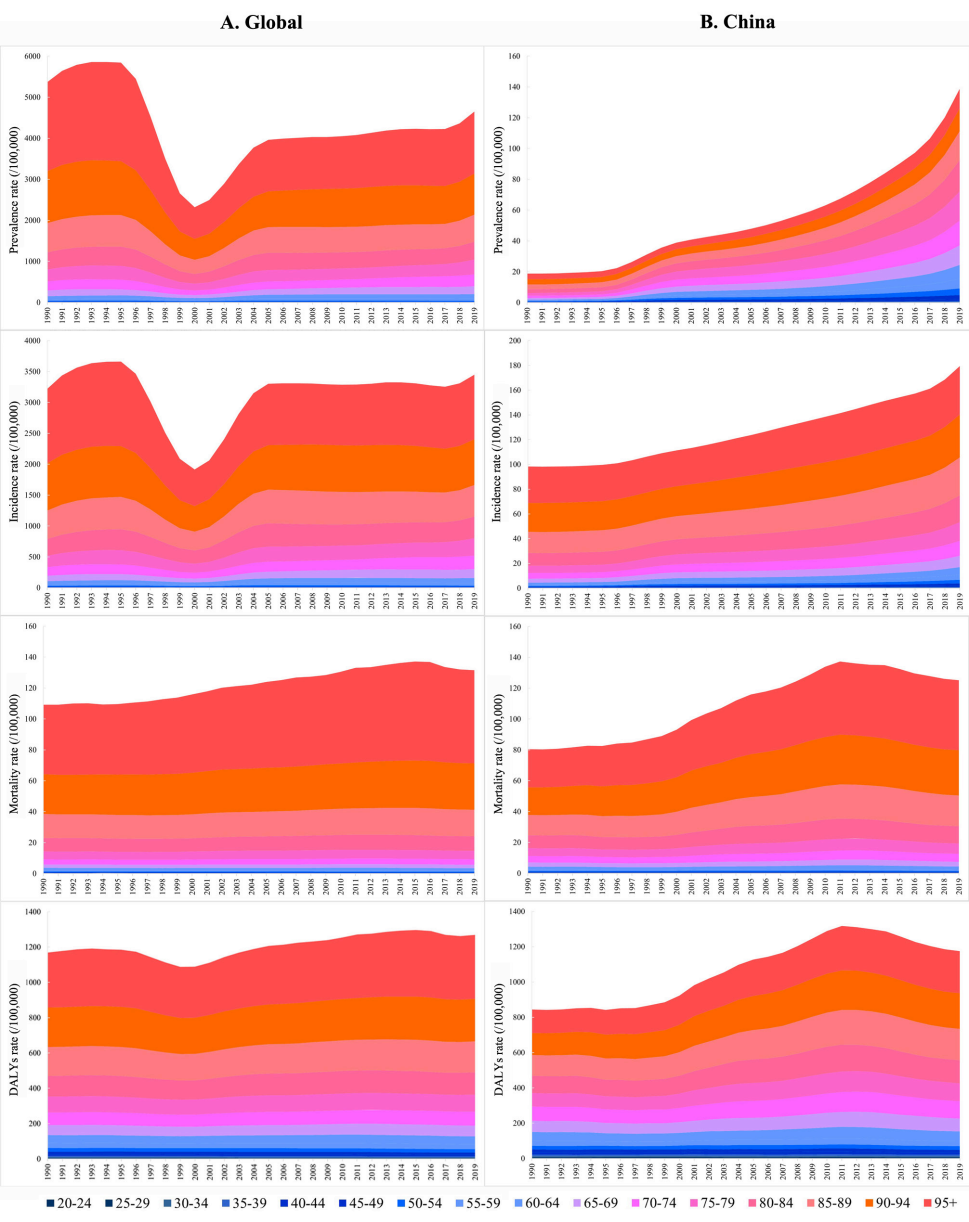
The age distribution of prevalence rate, incidence rate, mortality rate and DALY rate for cSCC from 1990 to 2019 in the global and China level. **Panel A.** Prevalence rate, incidence rate, mortality rate and DALYs rate for cSCC worldwide. **Panel B.** Prevalence rate, incidence rate, mortality rate and DALY rate for cSCC in China. DALY – disability-adjusted life years.

Globally, the cSCC burden indicators among the population over 20 years of age rose with age group each year and peaked in the ≥95 age group. In line with the gender distribution, the prevalence rate and incidence rate of cSCC for all age groups also reached the lowest point in 2000 and increased yearly afterwards. The prevalence and incidence rates of cSCC peaked in the ≥95 age group in 1995 at 2406.63 and 1367.56 per 100 000 population, respectively. Meanwhile, the mortality rate and DALYs rate of cSCC fluctuated relatively steadily from 1990 to 2019 and peaked in the ≥95 age group in 2016 and 2015, at 63.94 and 377.12 per 100 000 population, respectively (**Figure 4**).

In China, the prevalence rate of cSCC increased with age from 1990 to 1997 and peaked in the 80–84 age group from 1998 to 2019 (20.31 per 100 000 population in 2019). The incidence rate, mortality rate, and DALYs rate of cSCC increased with age each year, peaking in the ≥95 age group in 2019 (39.26 per 100 000 population), 2014 (47.56 per 100 000 population), and 2011 (251.78 per 100 000 population) (**Figure 4**).

### The prediction of disease burden of cSCC from 2020 to 2030

**Figure 5:**
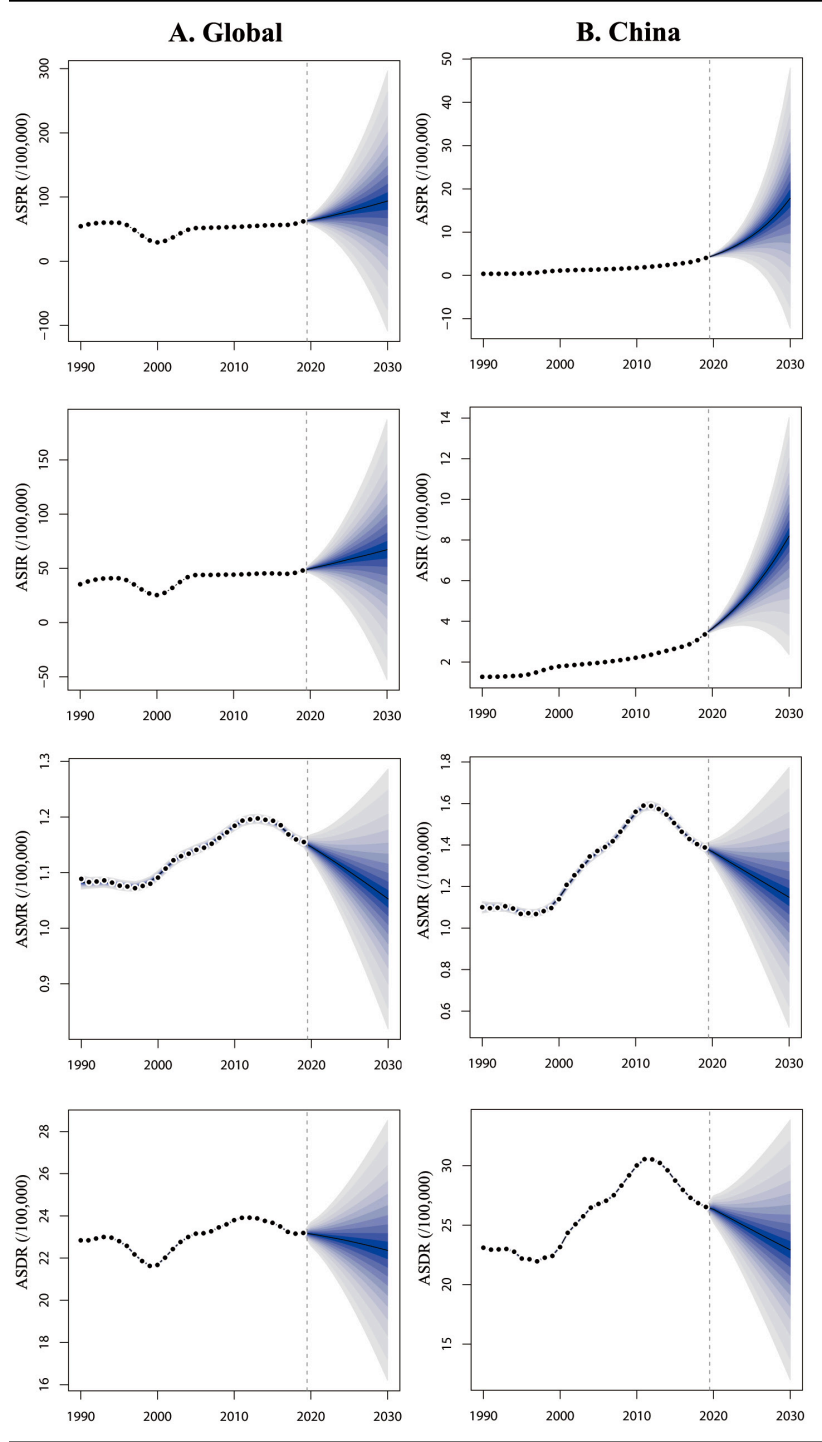
Prediction of ASIR, ASPR, ASMR and ASDR for cSCC worldwide and in China from 2020 to 2030. **Panel A.** ASIR, ASPR, ASMR and ASDR for cSCC worldwide. **Panel B.** ASIR, ASPR, ASMR and ASDR for cSCC in China. ASDR – age-standardised disability-adjusted life years rate, ASMR – age-standardised mortality rate, ASIR – age-standardised incidence rate, ASPR – age-standardised prevalence rate.

The predictions based on the BAPC model indicated that there will be an upward trend in the prevalence and incidence of cSCC globally and in China between 2020 and 2030. However, they projected a decreasing trend in mortality and DALY rates. Specifically, in 2030, the global ASPR and ASIR are projected to reach 59.28 and 42.44 per 100 000 population, respectively, representing increases of 50.88% and 40.07% compared to 2019. Meanwhile, the ASMR and ASDR are expected to decrease to 0.67 per and 14.13 per 100 000 population, respectively, representing reductions of 8.22% and 3.48% compared to 2019 (**Figure 5**).

Within China, the ASPR and ASIR are projected to increase to 11.26 and 5.17 per 100 000 population, representing significant increases of 343.30% and 143.87% compared to 2019. Conversely, the ASMR and ASDR are expected to decrease to 0.73 and 14.49 per 100 000 population, respectively, representing reductions of 17.05% and 13.54% compared to 2019 (**Figure 5**). Furthermore, the projections suggested that the disease burden of cSCC will be more significant among the elderly population worldwide and in China between 2020 and 2030 (Tables S2–10 in the **Online Supplementary Document**).

### Sensitivity analyses

Despite significant fluctuations in the estimates in the sensitivity analyses, the overall findings remained stable and the BAPC model remained reliable for predicting the disease burden of cSCC. Based on the calculated coefficient of determination (R^2^), it showed consistency in its predictive ability (Table S11 in the **Online Supplementary Document**).

## DISCUSSION

Based on data from the GBD 2019 study, we analysed the trend of the cSCC burden in China and compared it with the global trends from 1990 to 2030. Our findings suggest that the global cSCC burden remained stable for the past three decades. Conversely, although Chinese prevalence and incidence rates were relatively low, they increased faster than the global average. As it has the world’s largest population, China needs such accurate data to effectively conduct public health surveillance and interventions.

Globally, the changing trend of the cSCC burden has varied broadly across different countries. For example, high-SDI countries with a large population of light-skinned people such as Canada, America, and Australasia had the highest ASRs for cSCC burden. Most Asian and African regions with middle- or low-SDI countries and coloured populations reported low ASRs. Research has shown that people with darker skin tones tend to have a lower rate of cSCC, while non-Hispanic white individuals usually have the highest incidence [18]. Moreover, while more than half of the Chinese participants (52.0%) in a recent study viewed tanning as harmful or unattractive [19], 90% of Irish people indicated in surveys that they believed sunbathing made them look healthier and more attractive [20,21]. We also found that areas with a high SDI have significantly higher ASIR and ASPR. High-SDI countries have a greater capacity for integrated health care services and can provide a higher level of care. For example, the American Academy of Dermatology has been sponsoring free volunteer dermatologist skin cancer education and screening programmes since 1985, providing over two million free screenings [22]. Meanwhile, health care systems in low- to middle-SDI countries face challenges resulting from economic disparities, inadequate funding for health care services, limited access to advanced health care technology, insufficient disease awareness, and inadequate health insurance coverage [7]. A study of 84 030 respnses from the National Health Interview Survey found that Asian Americans are more likely than non-Hispanic white Americans to seek shade and wear protective clothing, resulting in a lower incidence of sunburn and skin cancer [23]. Asian immigrant populations are predominantly young, well-educated, and economically affluent, which may contribute to increased population ageing and cSCC disease burden in countries of origin [23]. Although the ASPR and ASIR for cSCC in China were substantially below global levels in 1990–2019, both the ASPR and the ASIR for the cSCC burden indicators exhibited a significant and higher rate of increase than global levels.

Notably, China launched a USD 125 billion health care reform plan in 2009. In 2020, it managed to achieve almost universal health care insurance and established a foundation for primary health care and public health [24]. Moreover, the government recognised the ‘Healthy China’ plan as a basic state policy in 2016, prompting greater health awareness and improved health care, resulting in higher rates of outpatient visits and detection of cSCC [25]. This is possibly the reason why the incidence rate of cSCC exceeded the mortality rate, leading to a significant increase in the prevalence rate.

Meanwhile, the ASRs for men were higher than those for women globally and in China from 1990 to 2019. We also observed a divergence in the direction of the trend for the ASRs of men and women at various points. For instance, we saw more significant increases in the incidence of cSCC among men than women, both in China and globally. Moreover, most Chinese women believe that fair skin can make them more attractive [26]. Meanwhile, a study of 623 individuals from northern China found differences between men and women in their knowledge, awareness, and behaviour towards sun protection, where the women scored significantly higher in these areas (*P* = 0.0001), while 16.4% of men reported not using sun protection methods [19]. In line with this, a survey of 5964 individuals in Shanghai found that over 70% of men did not wear hats or long pants and long-sleeved shirts to avoid the sun, 88% never used umbrellas, and 97% never used sunscreen [27,28], while Kimlin et al. [29] found that men in China experience notably greater UV exposure due to extensive outdoor activities and little sun protection.

Certain countries categorise cSCC as an occupational disease affecting outdoor workers, especially men, with 47% of these workers not having seen a dermatologist [30]. Men may also be more likely to delay screening or diagnosis of asymptomatic illnesses and may be less concerned about the possibility of recurrence. Additionally, Kakagia et al. [31] reported that men had a 41.7% delay in presentation for cSCC, almost twice that which was observed for women. However, only a few European countries have implemented specific prevention, screening, and follow-up programmes for occupational skin cancer. Gender-based disparities in sun-protective behaviours (such as sunscreen application or wearing sun-protective clothing), outdoor work, and attitudes toward disease management could explain the differences in the burden of cSCC.

The ASPR and ASIR of cSCC for both genders kept increasing from 1990 to 2019, while the ASMR and ASDR gradually increased and peaked in 2012 and subsequently decreased annually. Since 2005, China has organised a Skincare Day annually on 25 May to promote awareness of scientific skincare and sun protection [32]. The best protective way is to minimise sun exposure, especially between 10 am to 2 pm It is also advisable to wear sunglasses, long-sleeved clothing and a hat, and regularly apply a sunscreen cream with a high sun protection factor. With the rapid growth of the Chinese economy in the past decade, sun protection has become affordable for more Chinese [19]. However, a significant number of individuals still fail to use sunscreen products regularly or correctly [33]. Moreover, most people in China rely on non-medical sources for medical information. Among parents, 52% see TV commercials as a significant way to promote sun protection. To ensure adequate skin protection, medical professionals should be involved in the creation of these commercials [33]. Recent data on ASRs and substantial health care investments in China suggest that a successful strategy emphasising public awareness and early detection can lead to the identification of more early cases of cSCC and a subsequent reduction in mortality rates [34]. Similarly, Zink et al. [30] suggested that national governments collaborating with professional organisations and providing tailored preventive measures for various types of outdoor workers to be the most effective approach to reducing the burden of skin cancer. Various intervention strategies are needed, such as training new employees, organising outdoor work schedules, and supplying protective clothing or sunscreen. Likewise, regular educational and assessment initiatives focussed on sun protection information and disease screening could support and benefit the high-risk population identified for cancer.

Consistent with previous reports, our data indicate that the incidence of cSCC rises with age [35]. The trend highlights the impact of an ageing population on the disease burden of cSCC and underlines the importance of age standardisation when comparing incidence rates across different populations. Specifically, the incidence of cSCC has been reported to be 5 to 10 times higher in people over 75 years of age than in people under 55, and as much as 50 to 300 times higher in people under 45 [35–37]. Moreover, a survey of residents over 60 in a community in Shanghai, China, found that skin cancer tended to increase with age, especially in groups over 75 years old [38]. Patients with cSCC may exhibit more chronic sun injury, leading to experiencing multiple cSCCs across their lives, which could be linked to specific patterns of total and cumulative UV exposure [39]. Yan et al. [28] showed that sun exposure and attitudes toward sunbathing varied among Chinese, with the most notable disparities related to gender and age. Sun protection measures are often neglected by men and elderly individuals, with older people having a comparatively lower understanding and usage of sunscreen knowledge. Moreover, lesions that are detected early may be cured by surgical removal with a good prognosis, while those detected late at an older age are significantly more likely to metastasize and may require extensive surgery, resulting in a poorer prognosis. Additionally, older individuals experience a decline in physical function and are susceptible to multiple underlying diseases. It has been suggested that older people with hypertension or diabetes may have a higher risk of skin tumours, possibly because of the photosensitive characteristics of antihypertensive drugs or the persistent hyperglycaemic state experienced by diabetic patients [37–40].

In 2019, there were 164.5 million Chinese citizens aged 65 years and above, of whom 26 million were aged 80 and above. It is estimated that by 2050, the number of people aged 65 and over could increase to 450 million, and therefore account for more than one-third of the total population. Given the ageing population, it is imperative to introduce corresponding health insurance and policy adjustments to ease the burden on this demographic group. To further enhance primary health care systems and provide training for caregivers and medical professionals, China has established the National Clinical Research Center for Geriatric Disorders [41], aiming to promote health equity among older individuals. We also observed another trend in the distribution of the burden by age and gender, where the ASPR and ASIR of cSCC reached their lowest point in 2000 and have been increasing steadily each year since. Cancer-related policies in the United States may have contributed to this trend, as our study showed that changes in the data from the USA significantly affect global trends (Table S1 in the **Online Supplementary Document**).

The predictions based on the BAPC model indicated an upward trend in the prevalence and incidence of cSCC globally and in China between 2020 and 20230. The incidence trends were mainly driven by age, particularly in the elderly population. The large and populous country of China and the global ageing population may contribute to this phenomenon. Cancer incidence generally increases with age, particularly with epithelial cancer, where the risk amplifies by approximately five to six times with age, resulting in a difference of up to 1000 times in cancer rates between young and older people [42]. Another possible reason is the impact of increased screening and improved diagnostic pathways which have allowed for the detection of cases that may have previously gone undiagnosed. The visual nature of dermatology makes it highly suitable for online consultation, and its effectiveness and reliability have been successfully demonstrated. China is emerging as the leading market for telehealth consultations over the Internet, with diagnostic accuracy ranging from 68% to 87% [43]. Moreover, various environmental and social changes have the potential to result in alterations to data by 2030. Increased levels of UV radiation (UVR) caused by stratospheric ozone depletion can cause DNA damage to keratinocyte cells, which is strongly associated with the development of cSCC. Increased temperatures caused by global warming can lead to increased time spent outdoors and less clothing, which alters the diversity of microorganisms on the skin and exacerbates inflammation caused by exposure to UVR [44]. However, a limited number of studies has made it challenging to develop suitable policies for the Chinese population concerning understanding the impact of UVR doses on skin health. Specifically, some studies used satellite measurements of cloud-adjusted ambient UVR at different latitudes, while others disagreed with this approach. Personal UVR is influenced not only by environmental factors but also by behavioural factors such as time spent outdoors and sun protection [29]. Collecting precise personal UVR dosimetry, along with information on gender, age, lifestyle, education, and occupation, will enable the customisation of preventive measures.

Conversely, the model projected a decrease in mortality and DALY rates in the next decade, likely attributable to more standardised diagnostic guidelines and real advancements in treatment regimens. However, these trends are expected to vary considerably between countries worldwide, possibly due to factors such as health expenditure, public health policies, and treatment access. The National Natural Science Foundation of China has provided 10 million dollars of funding for over 100 skin research projects, recognising the significance of skin research for the nation's health [45]. As of December 2015, China has 22 000 dermatologists, while the related dermatology and skin biology research is rapidly growing. The progress in dermatology in China, including a focus on prevention, improved diagnostic tools, and optimised treatment strategies, has reduced the mortality and DALYs for cSCC.

The HAQ index serves as a crucial measure for monitoring the health service sector. Among middle-SDI countries, China has made significant progress in HAQ since 1990. However, its HAQ index for cSCC was relatively low, ranking last globally [7]. Although the disease burden of cSCC is not currently severe, the growing trend requires further investigation. The changing trends of cSCC, especially the problems caused by ageing, will challenge the physical health and economic status of the elderly, while also straining the health care system. A thorough investigation into China’s elderly population and the implementation of health care policy reforms are crucial. Enhancing primary health care services, advocating for medical education, improving health care accessibility and quality, and delivering top-notch health care services are all indispensable in raising China's HAQ index and lessening the overall disease burden. Given its status as a representative developing nation, China’s health care reform strategy can provide valuable lessons for other countries with similar characteristics and serve as a blueprint for those aspiring to establish universal health insurance.

Some limitations of our study should be considered. First, despite efforts to mitigate data bias through adjusted methods in the GBD 2019 and confirmation of reliability through previous studies, these data are not measured directly and may still be subject to bias due to the difficulty in determining the underlying cause of death, missing and lagging census data in some areas, and other reasons [8]. Second, the GBD 2019 study also lacked data for urban/rural distribution, specific Chinese provinces, and risk factors of cSCC. This prevents more in-depth research, which can be supplemented by accurate surveillance data in the future. Third, due to the unavailability of relevant data, our predictions could not account for the impact of changes in external factors such as health care policy, public health initiatives, or economic fluctuations on the burden of cSCC. Fourth, the actual prevalence of cSCC is uncertain due to lacking reporting in cancer registries. Epidemiological research typically combines data for cSCC, basal cell carcinoma, and other non-melanoma skin tumours, leading to notable discrepancies in estimated incidence rates. Lastly, disparities in health care systems regarding financial allocation and administration can result in differences that hinder the comparability of trends across countries.

## CONCLUSIONS

We observed similarities and differences in the burden of cSCC between China and the world. The prevalence of cSCC in China has noticeably increased over the last 30 years and is expected to continue to do so at a higher rate than the worldwide average in the next 10 years. Given the significant differences in geographic location, economic growth, and lifestyles across China’s provinces, more research is needed to provide data on the burden of CSCC in this context. Our findings suggest that, while there has been a gradual reduction in the burden of cSCC, it remains disproportionately higher burden among older individuals, suggesting a need to target management and prevention efforts at this population. Raising awareness of public education, enhancing screening programmes, promoting self-examination, improving treatment quality, and reducing sun exposure could help reduce the burden of cSCC in China.

## Additional material


Online Supplementary Document
