# Supplementary material for: Global burden and prediction study of cutaneous squamous cell carcinoma from 1990 to 2030: A systematic analysis and comparison with China
Source: J Glob Health. 2024 May 3;14:04093. doi: 10.7189/jogh.14.04093 (PMC11063968; doi:10.7189/jogh.14.04093)
Supplement: Online Supplementary Document [file jogh-14-04093-s001.pdf]

Table S1. Global ASPR, ASIR, ASMR and ASDR of cSCC by region in 2019

Region	ASPR (95%UI)	ASIR (95%UI)	ASMR (95%UI)	ASDR (95%UI)
High-income Asia Pacific	1.18 (0.97, 1.41)	1.21 (0.99, 1.44)	0.36 (0.30, 0.40)	6.00 (5.37, 6.42)
Central Asia	3.29 (2.83, 3.82)	2.91 (2.52, 3.29)	0.83 (0.70, 0.91)	15.57 (13.31, 17.14)
East Asia	2.47 (2.08, 2.92)	2.08 (1.76, 2.42)	0.87 (0.74, 0.99)	16.60 (14.11, 19.12)
South Asia	0.16 (0.13, 0.20)	0.38 (0.33, 0.44)	0.35 (0.27, 0.42)	6.53 (5.02, 7.76)
Southeast Asia	0.38 (0.32, 0.45)	0.88 (0.79, 0.98)	0.78 (0.67, 0.87)	15.66 (13.52, 17.62)
Australasia	275.86 (232.49, 327.40)	249.75 (210.03, 294.74)	1.70 (1.48, 1.85)	38.68 (34.11, 44.15)
Caribbean	2.62 (2.29, 3.00)	2.97 (2.68, 3.29)	1.41 (1.19, 1.63)	25.72 (21.65, 29.74)
Central Europe	4.78 (4.21, 5.42)	4.52 (4.07, 4.99)	1.08 (0.94, 1.21)	17.01 (15.00, 19.15)
Eastern Europe	2.84 (2.38, 3.37)	2.93 (2.44, 3.48)	0.86 (0.76, 0.96)	17.09 (15.12, 19.1)
Western Europe	8.16 (7.09, 9.51)	6.87 (5.93, 7.89)	0.60 (0.52, 0.64)	9.90 (9.04, 10.44)
Andean Latin America	2.80 (2.41, 3.29)	2.65 (2.31, 3.00)	1.06 (0.87, 1.27)	18.10 (14.53, 21.84)
Central Latin America	5.50 (4.78, 6.36)	5.07 (4.39, 5.81)	1.23 (1.04, 1.40)	21.46 (18.44, 24.75)
Southern Latin America	12.21 (10.66, 14.11)	9.86 (8.61, 11.16)	0.94 (0.83, 1.01)	16.10 (14.57, 17.05)
Tropical Latin America	4.49 (3.83, 5.28)	5.07 (4.29, 5.91)	1.22 (1.05, 1.31)	22.10 (19.88, 23.36)
High-income North America	427.07 (364.37, 504.72)	324.18 (285.94, 368.52)	0.75 (0.66, 0.79)	30.91 (25.05, 38.75)
Oceania	0.23 (0.19, 0.28)	0.71 (0.65, 0.78)	0.80 (0.65, 0.98)	14.56 (11.53, 18.65)
North Africa and Middle East	0.96 (0.82, 1.12)	1.15 (1.01, 1.30)	0.54 (0.47, 0.61)	9.08 (8.11, 10.14)
Central Sub-Saharan Africa	0.59 (0.49, 0.71)	0.85 (0.75, 0.98)	0.57 (0.36, 0.75)	10.62 (6.98, 13.89)
Eastern Sub-Saharan Africa	0.59 (0.49, 0.71)	0.87 (0.76, 1.00)	0.66 (0.34, 0.84)	11.66 (6.54, 14.69)
Southern Sub-Saharan Africa	6.71 (5.58, 8.11)	6.51 (5.29, 7.82)	0.86 (0.77, 0.93)	15.72 (14.19, 17.00)
Western Sub-Saharan Africa	0.31 (0.25, 0.37)	0.48 (0.42, 0.56)	0.38 (0.29, 0.44)	7.71 (5.90, 9.26)

ASDR - age-standardized disability-adjusted life-years rate, ASIR - age-standardized incidence rate, ASMR - age-standardized mortality rate, ASPR - age-standardized prevalence rate, cSCC - cutaneous squamous cell carcinoma.

Table S2. The predicted age-standardized rates (per 100,000 population) in prevalence, incidence, mortality and DALYs by age group for cSCC from 2020 to 2030

Years	Global				China			
	**Prevalence**	**Incidence**	**Mortality**	**DALYs**	**Prevalence**	**Incidence**	**Mortality**	**DALYs**
2020	31.43	0.72	40.29	14.62	2.31	0.87	2.91	16.64
2021	32.43	0.72	41.89	14.58	2.50	0.86	3.34	16.42
2022	33.43	0.71	43.57	14.54	2.71	0.84	3.83	16.20
2023	34.50	0.71	45.36	14.50	2.94	0.83	4.39	15.99
2024	35.64	0.70	47.24	14.46	3.19	0.81	5.04	15.77
2025	36.79	0.70	49.16	14.41	3.46	0.80	5.77	15.55
2026	37.89	0.69	51.05	14.36	3.75	0.79	6.60	15.33
2027	38.96	0.68	52.98	14.30	4.06	0.77	7.55	15.12
2028	40.07	0.68	55.00	14.25	4.41	0.76	8.63	14.91
2029	41.25	0.67	57.12	14.19	4.77	0.74	9.86	14.70
2030	42.44	0.67	59.28	14.13	5.17	0.73	11.26	14.49

cSCC - cutaneous squamous cell carcinoma, DALYs - disability-adjusted life years.

Table S3. The predicted prevalence rates (per 100,000 population) by age groups for cSCC from 2020 to 2030 globally

Years	Global ASPR																			
	**0-4**	**5-9**	**10-14**	**15-19**	**20-24**	**25-29**	**30-34**	**35-39**	**40-44**	**45-49**	**50-54**	**55-59**	**60-64**	**65-69**	**70-74**	**75-79**	**80-84**	**85-89**	**90-94**	**95+**
2020	0.00	0.00	0.00	0.00	0.15	0.32	0.80	2.02	4.85	11.19	24.65	56.92	115.97	196.46	297.29	374.26	444.35	646.52	965.94	1449.63
2021	0.00	0.00	0.00	0.00	0.16	0.34	0.84	2.08	5.08	11.52	25.32	57.12	120.70	205.81	309.33	400.79	474.04	670.12	994.80	1488.52
2022	0.00	0.00	0.00	0.00	0.16	0.35	0.88	2.16	5.32	12.00	26.19	57.55	124.88	217.77	322.16	430.63	513.25	702.41	1033.67	1537.39
2023	0.00	0.00	0.00	0.00	0.17	0.37	0.94	2.26	5.61	12.69	27.28	58.69	129.09	233.23	338.63	464.16	563.93	747.06	1084.67	1601.73
2024	0.00	0.00	0.00	0.00	0.18	0.39	1.00	2.41	5.95	13.62	28.68	60.99	134.16	252.71	361.37	502.34	626.02	807.49	1150.23	1685.90
2025	0.00	0.00	0.00	0.00	0.19	0.42	1.09	2.61	6.38	14.84	30.59	64.81	140.73	276.37	392.15	547.23	700.15	888.32	1234.96	1798.28
2026	0.00	0.00	0.00	0.00	0.21	0.46	1.20	2.88	6.94	16.41	33.26	70.28	149.08	303.64	433.67	601.06	791.49	1000.39	1351.23	1955.03
2027	0.00	0.00	0.00	0.00	0.24	0.51	1.33	3.25	7.70	18.40	37.03	77.68	160.54	335.74	490.42	669.02	908.85	1157.58	1513.68	2171.03
2028	0.00	0.00	0.00	0.00	0.27	0.58	1.51	3.73	8.73	20.96	42.36	87.56	177.19	375.65	568.49	761.14	1060.31	1376.63	1742.48	2465.78
2029	0.00	0.00	0.00	0.00	0.32	0.67	1.76	4.39	10.19	24.38	49.86	100.98	201.94	428.17	675.54	890.80	1258.50	1675.99	2065.58	2867.67
2030	0.00	0.00	0.00	0.00	0.38	0.81	2.10	5.29	12.29	29.09	60.44	119.75	238.60	499.42	821.50	1074.92	1524.45	2084.30	2526.74	3423.63

ASPR - age-standardized prevalence rate, cSCC - cutaneous squamous cell carcinoma.

Table S4. The predicted incidence rates (per 100,000 population) by age groups for cSCC from 2020 to 2030 globally

Years	Global ASIR																			
	**0-4**	**5-9**	**10-14**	**15-19**	**20-24**	**25-29**	**30-34**	**35-39**	**40-44**	**45-49**	**50-54**	**55-59**	**60-64**	**65-69**	**70-74**	**75-79**	**80-84**	**85-89**	**90-94**	**95+**
2020	0.01	0.00	0.00	0.00	0.17	0.31	0.81	1.76	4.36	8.93	19.29	40.56	85.03	146.31	228.75	302.01	374.03	526.45	752.58	1070.21
2021	0.01	0.00	0.00	0.00	0.18	0.32	0.83	1.79	4.50	9.07	19.58	40.22	87.43	151.57	235.38	320.00	395.91	541.85	769.11	1092.55
2022	0.01	0.00	0.00	0.00	0.18	0.33	0.87	1.83	4.64	9.31	19.95	39.96	89.18	158.19	241.80	339.06	423.96	562.52	790.95	1119.25
2023	0.01	0.00	0.00	0.00	0.19	0.34	0.91	1.89	4.80	9.65	20.42	40.06	90.65	166.63	250.01	359.21	459.25	590.95	819.41	1153.34
2024	0.01	0.00	0.00	0.00	0.20	0.36	0.95	1.98	4.99	10.13	21.02	40.78	92.36	176.99	261.74	381.05	501.08	629.20	855.84	1196.65
2025	0.01	0.00	0.00	0.00	0.21	0.37	1.01	2.09	5.22	10.73	21.85	42.27	94.60	188.95	277.69	405.72	548.77	679.50	902.35	1253.09
2026	0.01	0.00	0.00	0.00	0.22	0.40	1.08	2.24	5.51	11.49	23.03	44.51	97.36	201.62	298.55	433.27	603.44	746.42	963.84	1329.00
2027	0.01	0.00	0.00	0.00	0.24	0.43	1.16	2.44	5.91	12.41	24.73	47.48	101.22	215.21	326.07	465.78	669.09	836.45	1047.13	1430.27
2028	0.01	0.00	0.00	0.00	0.26	0.47	1.27	2.69	6.44	13.55	27.08	51.28	107.09	230.90	362.49	508.28	748.15	956.30	1161.00	1563.86
2029	0.01	0.00	0.00	0.00	0.29	0.52	1.41	3.01	7.15	14.98	30.24	56.21	116.07	250.46	409.93	566.53	844.97	1110.87	1316.09	1739.02
2030	0.01	0.00	0.00	0.00	0.34	0.59	1.59	3.43	8.13	16.83	34.43	62.75	129.27	275.61	470.17	645.75	966.56	1307.03	1526.96	1969.83

ASIR, age-standardized incidence rate; cSCC, cutaneous squamous cell carcinoma

TABLE S5 The predicted mortality rates (per 100,000 population) by age groups for cSCC from 2020 to 2030 globally

Years	Global ASMR																			
	0-4	5-9	10-14	15-19	20-24	25-29	30-34	35-39	40-44	45-49	50-54	55-59	60-64	65-69	70-74	75-79	80-84	85-89	90-94	95+
2020	0.00	0.00	0.00	0.00	0.03	0.04	0.07	0.11	0.18	0.28	0.48	0.76	1.43	2.24	3.53	5.45	9.38	17.00	30.08	60.00
2021	0.00	0.00	0.00	0.00	0.03	0.04	0.07	0.11	0.18	0.28	0.47	0.75	1.41	2.21	3.51	5.43	9.32	16.88	29.97	59.92
2022	0.00	0.00	0.00	0.00	0.03	0.04	0.07	0.11	0.18	0.27	0.47	0.75	1.39	2.18	3.49	5.41	9.26	16.78	29.83	59.84
2023	0.00	0.00	0.00	0.00	0.03	0.04	0.06	0.11	0.18	0.27	0.46	0.74	1.37	2.15	3.47	5.39	9.20	16.68	29.68	59.74
2024	0.00	0.00	0.00	0.00	0.03	0.04	0.06	0.11	0.17	0.26	0.45	0.73	1.35	2.11	3.44	5.38	9.15	16.58	29.51	59.63
2025	0.00	0.00	0.00	0.00	0.03	0.04	0.06	0.10	0.17	0.26	0.44	0.72	1.34	2.08	3.41	5.36	9.12	16.48	29.33	59.48
2026	0.00	0.00	0.00	0.00	0.02	0.04	0.06	0.10	0.17	0.26	0.44	0.71	1.33	2.05	3.37	5.34	9.09	16.37	29.15	59.30
2027	0.00	0.00	0.00	0.00	0.02	0.04	0.06	0.10	0.17	0.25	0.43	0.70	1.31	2.02	3.33	5.31	9.07	16.27	28.99	59.06
2028	0.00	0.00	0.00	0.00	0.02	0.04	0.06	0.10	0.17	0.25	0.42	0.69	1.30	2.00	3.28	5.28	9.05	16.19	28.84	58.80
2029	0.00	0.00	0.00	0.00	0.02	0.04	0.06	0.10	0.16	0.25	0.42	0.68	1.28	1.98	3.23	5.24	9.03	16.12	28.69	58.51
2030	0.00	0.00	0.00	0.00	0.02	0.04	0.06	0.10	0.16	0.24	0.41	0.66	1.27	1.96	3.18	5.20	9.00	16.07	28.54	58.21

ASMR - age-standardized mortality rate, cSCC - cutaneous squamous cell carcinoma.

Table S6. The predicted DALYs rates (per 100,000 population) by age groups for cSCC from 2020 to 2030 globally

Years	Global ASDR																			
	**0-4**	**5-9**	**10-14**	**15-19**	**20-24**	**25-29**	**30-34**	**35-39**	**40-44**	**45-49**	**50-54**	**55-59**	**60-64**	**65-69**	**70-74**	**75-79**	**80-84**	**85-89**	**90-94**	**95+**
2020	1.19	0.00	0.00	0.00	1.81	2.49	3.92	5.90	9.01	12.72	19.48	27.54	44.56	60.38	78.78	96.50	126.59	175.51	241.89	356.54
2021	1.19	0.00	0.00	0.00	1.80	2.47	3.89	5.87	8.92	12.59	19.33	27.46	44.15	60.06	78.97	96.95	126.90	175.64	242.29	357.59
2022	1.18	0.00	0.00	0.00	1.79	2.45	3.86	5.85	8.84	12.46	19.17	27.37	43.83	59.64	79.07	97.44	127.26	175.86	242.60	358.62
2023	1.18	0.00	0.00	0.00	1.77	2.44	3.83	5.83	8.78	12.33	19.00	27.26	43.61	59.13	79.08	97.93	127.69	176.20	242.85	359.65
2024	1.17	0.00	0.00	0.00	1.77	2.42	3.80	5.81	8.73	12.20	18.84	27.13	43.48	58.58	78.97	98.40	128.20	176.64	243.08	360.69
2025	1.16	0.00	0.00	0.00	1.76	2.41	3.77	5.77	8.69	12.08	18.67	26.96	43.38	58.02	78.73	98.80	128.81	177.16	243.37	361.70
2026	1.16	0.00	0.00	0.00	1.75	2.40	3.74	5.73	8.66	11.98	18.50	26.77	43.30	57.54	78.38	99.12	129.52	177.74	243.75	362.61
2027	1.15	0.00	0.00	0.00	1.74	2.38	3.72	5.69	8.63	11.88	18.32	26.58	43.20	57.18	77.91	99.36	130.31	178.43	244.31	363.44
2028	1.15	0.00	0.00	0.00	1.73	2.37	3.71	5.65	8.61	11.81	18.15	26.38	43.08	56.96	77.34	99.48	131.13	179.25	245.08	364.24
2029	1.14	0.00	0.00	0.00	1.73	2.36	3.69	5.62	8.58	11.75	17.99	26.19	42.92	56.86	76.72	99.48	131.93	180.21	246.02	365.09
2030	1.14	0.00	0.00	0.00	1.72	2.35	3.67	5.59	8.55	11.72	17.84	26.00	42.73	56.82	76.10	99.34	132.67	181.35	247.12	366.08

ASDR - age-standardized disability-adjusted life years rate, cSCC - cutaneous squamous cell carcinoma, DALYs - disability-adjusted life years.

Table S7. The predicted prevalence rates (per 100,000 population) by groups for cSCC from 2020 to 2030 in China

**Years**	**China ASPR**																			
	**0-4**	**5-9**	**10-14**	**15-19**	**20-24**	**25-29**	**30-34**	**35-39**	**40-44**	**45-49**	**50-54**	**55-59**	**60-64**	**65-69**	**70-74**	**75-79**	**80-84**	**85-89**	**90-94**	**95+**
2020	0.00	0.00	0.00	0.00	0.05	0.09	0.25	0.68	1.53	3.10	4.80	6.37	9.86	15.20	19.35	22.32	23.20	21.18	17.18	15.11
2021	0.00	0.00	0.00	0.00	0.05	0.10	0.28	0.77	1.73	3.53	5.47	7.25	11.33	17.62	22.48	25.85	26.89	24.45	19.83	17.47
2022	0.00	0.00	0.00	0.00	0.06	0.11	0.32	0.87	1.96	4.03	6.26	8.28	13.05	20.47	26.19	30.06	31.29	28.38	22.97	20.29
2023	0.00	0.00	0.00	0.00	0.07	0.12	0.36	0.98	2.23	4.62	7.20	9.51	15.07	23.87	30.68	35.18	36.59	33.15	26.73	23.66
2024	0.00	0.00	0.00	0.00	0.08	0.14	0.41	1.11	2.54	5.32	8.34	11.00	17.48	27.96	36.17	41.48	43.06	39.01	31.30	27.77
2025	0.00	0.00	0.00	0.00	0.09	0.16	0.47	1.27	2.93	6.17	9.73	12.83	20.38	32.93	42.96	49.33	51.05	46.28	36.95	32.82
2026	0.00	0.00	0.00	0.00	0.10	0.19	0.54	1.47	3.40	7.22	11.47	15.10	23.97	39.11	51.48	59.22	61.11	55.45	44.09	39.16
2027	0.00	0.00	0.00	0.00	0.12	0.22	0.63	1.72	3.98	8.52	13.64	17.99	28.51	46.89	62.30	71.87	74.01	67.19	53.28	47.24
2028	0.00	0.00	0.00	0.00	0.14	0.26	0.75	2.04	4.72	10.16	16.40	21.71	34.35	56.83	76.22	88.33	90.88	82.45	65.31	57.69
2029	0.00	0.00	0.00	0.00	0.17	0.31	0.90	2.45	5.67	12.27	19.98	26.60	42.01	69.70	94.42	110.12	113.32	102.59	81.28	71.44
2030	0.00	0.00	0.00	0.00	0.21	0.38	1.10	2.99	6.93	15.05	24.72	33.12	52.22	86.64	118.58	139.48	143.70	129.70	102.82	89.91

ASPR - age-standardized prevalence rate, cSCC - cutaneous squamous cell carcinoma.

Table S8. The predicted incidence rates (per 100,000 population) by groups for cSCC from 2020 to 2030 in China

Years	China ASIR																			
	**0-4**	**5-9**	**10-14**	**15-19**	**20-24**	**25-29**	**30-34**	**35-39**	**40-44**	**45-49**	**50-54**	**55-59**	**60-64**	**65-69**	**70-74**	**75-79**	**80-84**	**85-89**	**90-94**	**95+**
2020	0.00	0.00	0.00	0.00	0.07	0.10	0.25	0.55	1.12	1.96	2.93	4.02	6.65	10.48	13.72	16.50	24.36	35.00	41.16	47.27
2021	0.00	0.00	0.00	0.00	0.08	0.11	0.27	0.59	1.20	2.11	3.16	4.35	7.26	11.46	14.96	17.88	26.44	37.75	44.23	50.76
2022	0.00	0.00	0.00	0.00	0.08	0.11	0.29	0.63	1.28	2.27	3.42	4.72	7.92	12.56	16.35	19.42	28.71	40.82	47.58	54.57
2023	0.01	0.00	0.00	0.00	0.09	0.12	0.30	0.67	1.37	2.45	3.70	5.12	8.63	13.76	17.90	21.14	31.19	44.24	51.23	58.72
2024	0.01	0.00	0.00	0.00	0.09	0.13	0.32	0.71	1.46	2.63	4.01	5.55	9.41	15.08	19.63	23.07	33.90	48.07	55.25	63.27
2025	0.01	0.00	0.00	0.00	0.10	0.14	0.35	0.76	1.57	2.83	4.34	6.03	10.25	16.53	21.56	25.24	36.89	52.35	59.74	68.28
2026	0.01	0.00	0.00	0.00	0.11	0.15	0.37	0.82	1.68	3.05	4.70	6.55	11.17	18.14	23.73	27.69	40.22	57.13	64.79	73.80
2027	0.01	0.00	0.00	0.00	0.12	0.16	0.40	0.87	1.80	3.29	5.10	7.13	12.19	19.93	26.17	30.46	43.98	62.46	70.53	79.91
2028	0.01	0.00	0.00	0.00	0.12	0.17	0.43	0.94	1.93	3.54	5.54	7.78	13.33	21.91	28.90	33.62	48.25	68.41	77.08	86.74
2029	0.01	0.00	0.00	0.00	0.13	0.18	0.46	1.01	2.08	3.82	6.02	8.50	14.60	24.11	31.98	37.22	53.16	75.07	84.54	94.45
2030	0.01	0.00	0.00	0.00	0.14	0.20	0.49	1.09	2.24	4.13	6.54	9.30	16.01	26.54	35.45	41.33	58.80	82.57	93.06	103.22

ASIR - age-standardized incidence rate, cSCC - cutaneous squamous cell carcinoma.

Table S9. The predicted mortality rates (per 100,000 population) by groups for cSCC from 2020 to 2030 in China

Years	China ASMR																			
	**0-4**	**5-9**	**10-14**	**15-19**	**20-24**	**25-29**	**30-34**	**35-39**	**40-44**	**45-49**	**50-54**	**55-59**	**60-64**	**65-69**	**70-74**	**75-79**	**80-84**	**85-89**	**90-94**	**95+**
2020	0.01	0.00	0.00	0.00	0.04	0.06	0.09	0.14	0.26	0.33	0.53	0.82	1.86	3.10	5.20	6.67	11.32	19.88	29.99	44.84
2021	0.01	0.00	0.00	0.00	0.04	0.06	0.09	0.14	0.26	0.32	0.52	0.81	1.81	3.02	5.14	6.61	11.19	19.64	29.68	44.61
2022	0.01	0.00	0.00	0.00	0.04	0.06	0.09	0.14	0.25	0.31	0.50	0.79	1.77	2.94	5.06	6.56	11.06	19.41	29.33	44.33
2023	0.01	0.00	0.00	0.00	0.04	0.06	0.09	0.13	0.24	0.31	0.49	0.78	1.73	2.86	4.98	6.51	10.94	19.19	28.98	44.04
2024	0.01	0.00	0.00	0.00	0.04	0.06	0.09	0.13	0.24	0.30	0.48	0.76	1.70	2.78	4.88	6.46	10.83	18.99	28.64	43.73
2025	0.01	0.00	0.00	0.00	0.04	0.06	0.08	0.13	0.23	0.29	0.47	0.74	1.67	2.71	4.78	6.40	10.74	18.80	28.33	43.41
2026	0.01	0.00	0.00	0.00	0.04	0.06	0.08	0.13	0.23	0.29	0.46	0.73	1.64	2.65	4.67	6.34	10.68	18.63	28.06	43.07
2027	0.01	0.00	0.00	0.00	0.04	0.06	0.08	0.12	0.22	0.28	0.45	0.71	1.62	2.59	4.56	6.27	10.63	18.49	27.84	42.73
2028	0.01	0.00	0.00	0.00	0.04	0.05	0.08	0.12	0.22	0.27	0.44	0.70	1.59	2.55	4.46	6.20	10.59	18.37	27.66	42.42
2029	0.01	0.00	0.00	0.00	0.04	0.05	0.08	0.12	0.21	0.27	0.43	0.69	1.57	2.52	4.36	6.11	10.57	18.29	27.52	42.15
2030	0.01	0.00	0.00	0.00	0.04	0.05	0.08	0.12	0.21	0.26	0.42	0.68	1.55	2.49	4.28	6.02	10.55	18.26	27.41	41.96

ASMR - age-standardized mortality rate, cSCC - cutaneous squamous cell carcinoma.

Table S10. The predicted DALYs rates (per 100,000 population) by groups for cSCC from 2020 to 2030 in China

Years	China ASDR																			
	0-4	5-9	10-14	15-19	20-24	25-29	30-34	35-39	40-44	45-49	50-54	55-59	60-64	65-69	70-74	75-79	80-84	85-89	90-94	95+
2020	1.53	0.00	0.00	0.00	3.20	3.93	5.33	7.63	12.65	14.43	20.56	27.44	51.94	73.32	101.27	102.41	134.59	180.94	213.82	242.20
2021	1.53	0.00	0.00	0.00	3.20	3.91	5.25	7.47	12.37	14.14	20.20	27.17	50.89	71.69	100.60	101.94	133.58	179.72	212.87	242.23
2022	1.53	0.00	0.00	0.00	3.20	3.90	5.19	7.32	12.09	13.85	19.83	26.89	50.04	70.01	99.65	101.61	132.62	178.57	211.76	242.11
2023	1.53	0.00	0.00	0.00	3.20	3.90	5.14	7.19	11.83	13.56	19.46	26.59	49.40	68.34	98.38	101.38	131.75	177.47	210.58	241.91
2024	1.54	0.00	0.00	0.00	3.21	3.90	5.11	7.07	11.58	13.29	19.10	26.26	48.91	66.75	96.83	101.18	131.03	176.40	209.44	241.65
2025	1.54	0.00	0.00	0.00	3.21	3.91	5.09	6.97	11.35	13.02	18.76	25.90	48.51	65.33	95.06	100.93	130.52	175.41	208.42	241.32
2026	1.55	0.00	0.00	0.00	3.22	3.92	5.08	6.89	11.14	12.76	18.42	25.51	48.16	64.17	93.19	100.51	130.24	174.53	207.55	240.87
2027	1.55	0.00	0.00	0.00	3.23	3.93	5.09	6.83	10.95	12.51	18.10	25.11	47.81	63.29	91.28	99.86	130.22	173.80	206.85	240.34
2028	1.56	0.00	0.00	0.00	3.25	3.94	5.10	6.79	10.80	12.28	17.79	24.73	47.45	62.70	89.41	98.95	130.39	173.29	206.31	239.85
2029	1.57	0.00	0.00	0.00	3.27	3.96	5.13	6.77	10.67	12.07	17.50	24.38	47.05	62.33	87.69	97.78	130.66	173.04	205.90	239.52
2030	1.58	0.00	0.00	0.00	3.29	3.99	5.15	6.77	10.56	11.88	17.22	24.05	46.61	62.11	86.22	96.44	130.93	173.15	205.68	239.45

ASDR - age-standardized disability-adjusted life years rate, cSCC - cutaneous squamous cell carcinoma, DALYs - disability-adjusted life years.

Table S11. The predicted age-standardized rates (per 100,000 population) in prevalence, incidence, mortality and DALYs for cSCC from 2011 to 2019.

Years	Global				China			
	Prevalence	Incidence	Mortality	DALYs	Prevalence	Incidence	Mortality	DALYs
2011	28.60	0.75	34.18	14.94	1.43	0.99	1.17	18.45
2012	28.83	0.75	34.54	15.01	1.47	1.01	1.24	18.75
2013	29.07	0.75	34.93	15.08	1.51	1.03	1.31	19.05
2014	29.31	0.76	35.34	15.15	1.55	1.05	1.38	19.34
2015	29.54	0.76	35.75	15.21	1.59	1.07	1.45	19.63
2016	29.76	0.77	36.14	15.27	1.64	1.10	1.52	19.91
2017	29.97	0.77	36.54	15.33	1.69	1.12	1.60	20.20
2018	30.20	0.77	36.97	15.39	1.74	1.14	1.68	20.48
2019	30.44	0.78	37.43	15.44	1.79	1.16	1.77	20.76
R^2	0.583	0.771	0.831	0.918	0.966	0.968	0.966	0.969

cSCC - cutaneous squamous cell carcinoma, DALYs - disability-adjusted life years.
